# Alveolar Antral Artery: Does its Diameter Correlate with Maxillary lateral wall Thickness in Dentate Patients?

**Published:** 2014-07

**Authors:** Amin Rahpeyma, Saeedeh Khajehahmadi, Parvin Amini

**Affiliations:** 1 *Oral and Maxillofacial Diseases Research Center, School of Dentistry, Mashhad University of Medical Sciences, Mashhad, Iran.*; 2 *Dental Research Center, School of Dentistry, Mashhad University of Medical Sciences, Mashhad, Iran.*; 3 *Dental Material Research Center ,School of Dentistry, Mashhad University of Medical Sciences, Mashhad, Iran.*

**Keywords:** Artery, Cone-Beam Computed Tomography, Maxillary Sinus

## Abstract

**Introduction:**

Knowledge of the presence of the alveolar antral artery in the lateral maxillary sinus wall is essential for surgeons who operate in this region. The purpose of this study was to investigate the correlation between alveolar antral artery diameter and lateral maxillary bony wall thickness in dentate patients.

**Materials and Methods:**

Thirty five Cone-Beam Computed Tomography (CBCT) scans from 35 dentate patients were selected in coronal sections in three locations; second premolar (P2), first molar (M1), and second molar (M2). The presence of the alveolar antral artery in each situation was determined and the bone thickness in the region of alveolar antral artery was measured perpendicular to the lateral wall of the maxilla.

**Results:**

The alveolar antral artery was present in 67.1% CBCTs. The difference between the alveolar antral artery diameter was only significant in the first and second molar area (P=0.039).There were significant differences between bone thickness in three locations, with the thickest bone in the first molar area followed by the second molar and second premolar, respectively. The correlation coefficient showed that there is a positive correlation between bone thickness and alveolar antral artery diameter.

**Conclusion:**

This study showed that the thicker the bones in dentate patients, the greater the chance of interference with the large caliber intra-osseous alveolar antral artery.

## Introduction

Anastomosing branches are located between the posterior superior alveolar artery (PSAa) and the infraorbital artery, both in the intra- and extra-osseous context. Intra-osseous anastomosis is known as the alveolar antral artery that gives branches to the Schneiderian membrane and maxillary periosteum (1). Extra-osseous anastomosis was shown by Traxler in a cadaveric study to be present in 44% of dissections (2).

Alveolar antral artery can be invaded on the lateral maxillary sinus wall during surgical procedures such as open sinus lift surgery, horizontal osteotomy of the maxilla, LeFort I fracture treatment, and Caldwell-Luc surgeries (3). As most of the research in this topic has focused on open sinus lift surgery (4), we decided to evaluate the prevalence of this artery with radiographic examinations (Cone-Beam Computed Tomography [CBCT]) of the lateral maxillary sinus wall and investigate the relationship between the diameter of the alveolar antral artery with the maxillary lateral wall thickness in dentate patients.

## Materials and Methods

**Fig 1:**
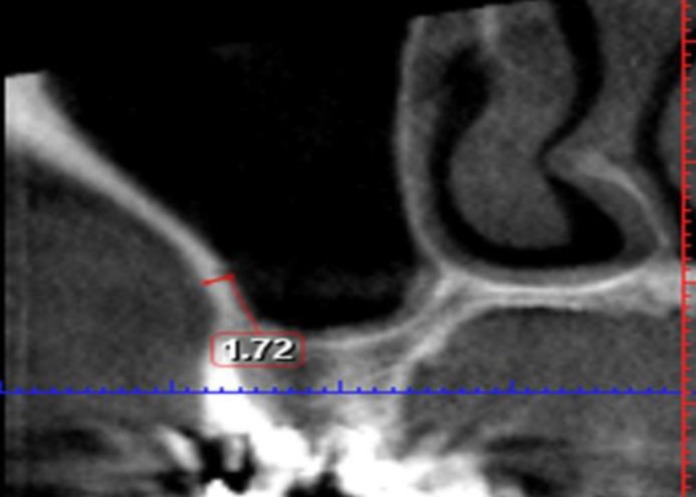
Alveolar antral artery in the lateral maxillary sinus wall

High quality CBCTs from the archive file were selected. The key inclusion criterion was presence of complete permanent dentition (with or without wisdom teeth), while the patient age was required to be 25–40 years. The criteria for exclusion were the presence of radiographic signs of sinusitis (acute or chronic), upper jaw fractures, and pathologic lesions that involve the maxillary sinus. All CBCTs were performed using by ProMax 3D (Planmeca Co., Helsinki, Finland) at 0.16-mm pixel resolution, 8 kvp, 8 mA, and 12 s. Using 2-mm thick reconstruction algorithms, the axial images were reconstructed into para-axial cross-sections. Coronal sections in three locations: second premolar (P2), first molar (M1) and second molar (M2) were chosen. The presence of the alveolar antral artery in each situation was determined. If no artery was present, it was recorded and measurements were discontinued. In the presence of a alveolar antral artery, its diameter was measured with a digital ruler using Romexis F software (Planmeca Romexis 2.4.2.R). The bone thickness in the region of the alveolar antral artery was measured perpendicular to the lateral wall of the maxilla in three locations, (P2, M1, and M2) (Fig.1). In the presence of two alveolar antral arteries in a coronal section, the larger one was measured. The measurements were carried out on the right and left sides.

## Results

A total of 35 CBCT scans were available with criteria suitable for this study. The majority of scans (55.7%) came from females. 

The alveolar antral artery was present in 67.1% of all scans. Table.1 shows the mean diameter of the artery in the three locations. Repeated measurements showed that the diameter of the artery in three locations was statistically different (P=0.027). 

**Table 1:** The diameter of alveolar antral artery in three locations

**location**	**Diameter of artery**
	**SD** ** ± ** **mean**
P2	314/0 ± 024/1
M1	320/0 ± 124/1
M2	311/0 ± 936/0
P value	027/0

**Fig 2:**
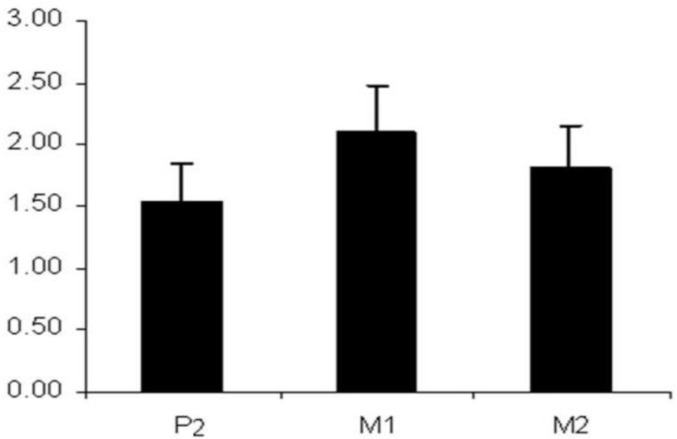
Bone thickness in three locations (second premolar, first molar, second molar) in millimeters

Table 2 shows the results of the analysis using a paired T test with a Bonferoni correction. The difference between alveolar antral artery diameter was significant only in the first and second molar area (P=0.039). No significant difference was identified between the right and left sides or between males and females. Analysis of the bone thickness is shown in (Table.3,Fig. 2). 

**Table2:** Comparison between the means of alveolar antral artery

**location**	**Mean difference**	**P value**
P2 , M1	101/0-	0/295
P2 , M2	061/0	0/852
M1 M2,	161/0	0/039

**Table3:** Bone thickness in three locations

**location**	**Bone thickness**
	**SD** ** ± ** **mean**
P2	0/311±1/528
M1	0/373 ± 2/096
M2	0/343± 1/799
P value	0/001>

**Fig 3:**
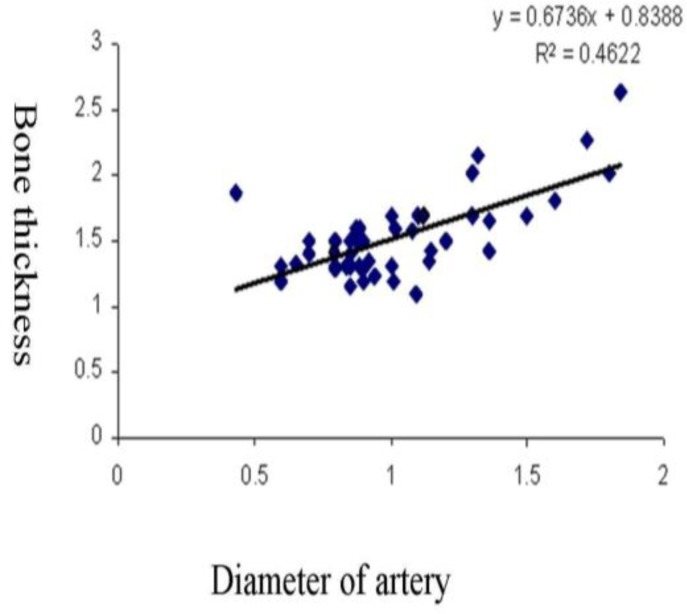
Regression curve between lateral maxillary bone thickness and alveolar

Significant differences among the three locations were identified, with the thickest bone in the first molar area followed by the second molar and second premolar respectively. For evaluation of the relationship between bone thickness and alveolar artery diameter, a Pearson correlation was applied to (P2,M1,M2) (Table.4), with the most positive correlation identified in the case of the second premolar (Fig. 3).

**Table 4:** Correlation between bone thickness and the diameter of alveolar antral artery.

**location**	**Correlation between bone thickness and diameter of artery**	
	correlation coefficient	P value
P2	0/68	0/001>
M1	0/303	0/04
M2	0/341	0/02

## Discussion

The alveolar antral artery is an anatomically important structure within the lateral maxillary sinus wall. The presence of this artery was first mentioned in an article published in 1934 (5). Severing this artery can lead to copious bleeding (6). Investigations into this artery can be divided into two groups:a) cadaveric studies and b) radiographic studies (Table.5). This artery is present in all cadaveric studies but in only 47–67% of radiographic studies (CBCT and CT scans) (7–10). The reasons for this may include 1) the fact that the small diameter arteries (<0.5 mm) are not detectable on CBCT or CT scans and 2) a number of alveolar antral arteries are subperiosteal, so they will not be detected on routine CBCTs.

**Table 5:** Summary of the previous works on alveolar antral artery

**References **	**Author** **year**	**Study design**	**Number**	**Presence of artery**	**Details**
7	Elian 2005	CT scan	50	53%	
8	Solar 1999	Cadaver	18	100%	Formalin/phenol fixed cadavers
9	Rosano 2010	CBCT	100	47%	
		Cadaver	15	100%	Formalin fixed cadavers
10	Mardinger 2007	CBCT	208	55%	
	Present study	CBCT	35	67%	
					

**Fig 4:**
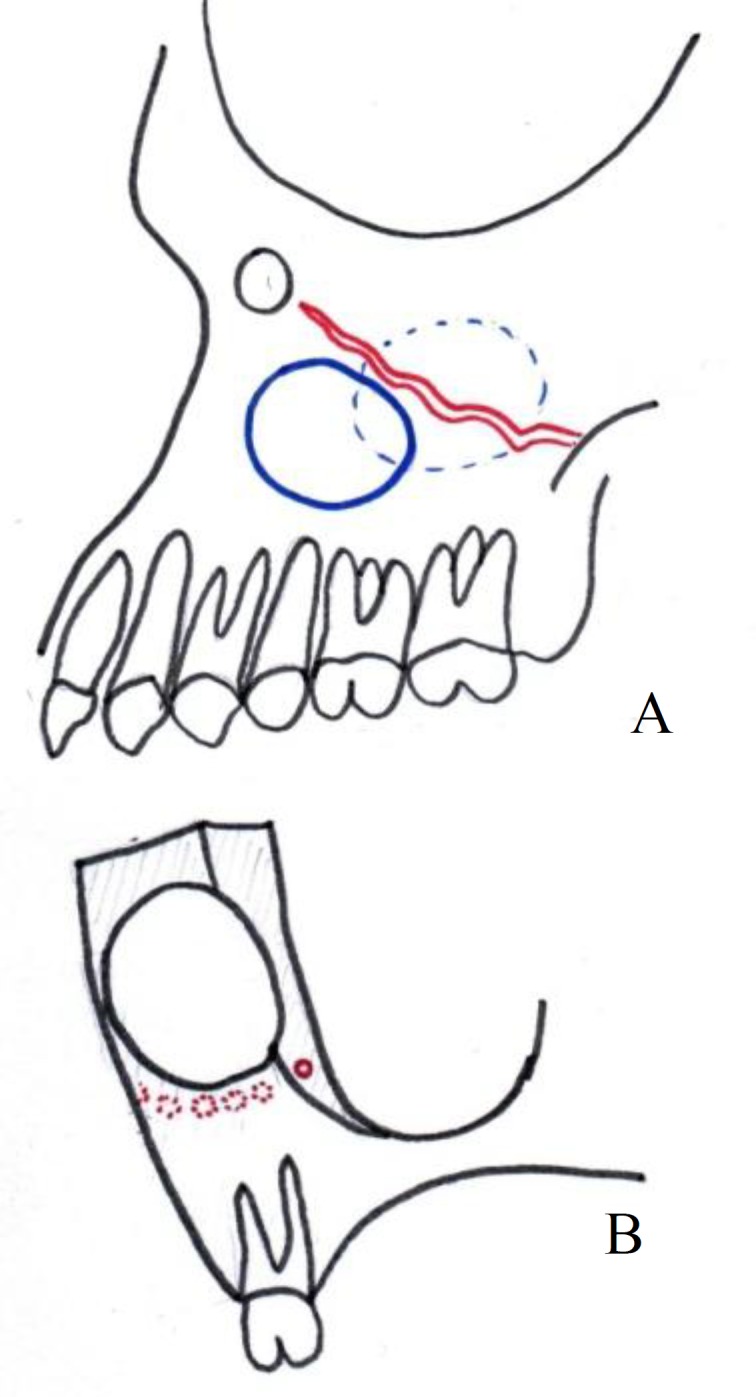
Ostectomy window can move to a place with less interference to alveolar antral artery. A, Anterior to the artery. B, Superior to the artery

Awareness of the diameter of this artery before surgery makes the surgeon more alert to the possible risk of hemorrhage during surgery and can change the bone cuts or location of the osteoctomy window (Fig.4). The greater the diameter of the artery, the greater the of risk hemorrhage (11). Management of bone hemorrhage during surgery is possible using electrocautery, bone wax or topical thrombin, or compression of the bone edges using hemostat beaks (12). In some cases, the presence of this artery in the lateral maxillary wall can be mistaken for a fracture.

The present study was performed in dentate patients because presence of teeth has an influence on the course and location of these vessels (13). The mean diameter of this artery has been measured by Kim as 1.52 ± 0.47mm (14), which is close to our results. Our results showed that surgery in the second molar area carries the least risk of interference with the large caliber alveolar antral artery. A study by Sato showed that calcitonin gene-related peptide (CRP) sensory nerve fibers accompany this artery (15), suggesting that injury to this vessel may increase post-operative pain. Preoperative evaluations of the lateral wall thickness and special attention to this artery can reduce intraoperative complications. Detection of this artery is possible using a waters view, CBCT or CT scan. The presence of this artery is not so obvious in plain radiographs, but it can be predicted that in a thicker bony wall, the surgeon can expect to confront a larger diameter alveolar antral artery. 

This artery represents an intra-osseous anastomosis between the infraorbital artery and the posterior superior alveolar artery (16). It participates in nourishing the Schneiderian membrane and has an important role in providing blood supply to the maxillary posterior teeth. Results of this study show that this artery does not have a constant diameter, and that its diameter depends on the thickness of the surrounding bony wall.

## Conclusion

This study showed that in dentate patients with thicker bones, there is a greater chance of interference with the large caliber intra-osseous alveolar antral artery.
